# Digital behavior patterns and ADHD symptoms in late adolescents and young adults

**DOI:** 10.3389/fpsyt.2026.1968257

**Published:** 2026-10-08

**Authors:** Shu Chen, Guangying He, Jingbo Zhao

**Affiliations:** 1 Office of Student Affairs, School of Public Health, Southern Medical University, Guangzhou, Guangdong, China; 2 Department of Psychology, School of Public Health, Southern Medical University, Guangzhou, Guangdong, China; 3 Mental Health Center, School of Public Health, Southern Medical University, Guangzhou, China

**Keywords:** ADHD symptoms, digital behavior patterns, fragmented information consumption, late adolescents, media multitasking, young adults

## Abstract

**Background:**

Digital media use has become deeply embedded in the daily lives of late adolescents and young adults, yet less is known about how specific digital behavior patterns are associated with ADHD symptom dimensions. This study focused on three emerging digital behaviors—speed watching, fragmented information consumption, and media multitasking—and examined their associations with inattention and hyperactivity-impulsivity within an ecologically informed multidomain framework encompassing demographic, perceived environmental, and family correlates.

**Methods:**

A total of 3,418 high school and university students aged 15–25 years (M_age = 18.60, SD = 1.05; 60.56% female) participated in the study, including 1,929 high school students (56.44%) and 1,489 university students (43.56%). They completed self-report measures of ADHD symptoms, digital behavior patterns, perceived physical environment, family environment, and demographic characteristics. ADHD symptoms were assessed in two dimensions: inattention and hyperactivity-impulsivity. Digital behavior patterns included speed watching, fragmented information consumption, and media multitasking. Pearson correlation analyses and hierarchical regression models were conducted to examine bivariate associations and the incremental explanatory value of digital behavior patterns after accounting for demographic, perceived environmental, and family variables.

**Results:**

Fragmented information consumption showed the strongest bivariate correlations with both inattention (r = 0.588) and hyperactivity-impulsivity (r = 0.488). In the hierarchical regression models, entering the digital behavior variables after the demographic, perceived environmental, and family variables accounted for an additional 22.3% of the variance in inattention and 14.5% of the variance in hyperactivity-impulsivity. Fragmented information consumption remained the strongest statistical correlate of both inattention (*β* = 0.379, *p* < 0.001) and hyperactivity-impulsivity (*β* = 0.256, *p* < 0.001), followed by media multitasking. The final models explained 44.0% and 34.9% of the variance in inattention and hyperactivity-impulsivity, respectively.

**Conclusion:**

Among the examined ecological correlates, digital behavior patterns—particularly fragmented information consumption and media multitasking—showed the most pronounced associations with ADHD symptoms in late adolescents and young adults. These findings suggest that distinguishing among specific forms of digital engagement may provide useful behavior-specific information about ADHD symptom variation. Because total screen time was not assessed, the findings do not establish that these behaviors are more informative than overall screen exposure. Given the cross-sectional and self-report design, the findings should be interpreted as associations rather than evidence of causal effects.

## 1 Introduction

Attention-deficit/hyperactivity disorder (ADHD) is a common neurodevelopmental condition characterized by developmentally inappropriate and impairing patterns of inattention, hyperactivity, and impulsivity (1). Although ADHD is usually identified during childhood, ADHD symptoms often remain clinically and functionally relevant during adolescence and young adulthood, a developmental period marked by increasing academic demands, greater autonomy, and intensive engagement with digital media. Recent evidence suggests that adolescents with mental health conditions show distinct patterns of social media use, underscoring the need to examine digital engagement as a behaviorally specific context rather than as a uniform exposure (2). In emerging adults, ADHD symptoms have also been linked to problematic forms of digital media use, including problematic social media use and problematic gaming (3). In non-clinical populations, self-reported symptoms of inattention and hyperactivity-impulsivity may therefore be meaningfully associated with difficulties in learning, self-regulation, daily functioning, and social adaptation. Accordingly, examining the behavioral correlates of ADHD symptoms in late adolescents and young adults may help clarify which everyday digital behavior patterns are most closely linked to attention- and impulse-related symptom variation.

Digital media use has become one of the most salient behavioral contexts in this developmental period. Prior longitudinal evidence suggests that frequent digital media use is associated with subsequent ADHD symptoms among adolescents (4), and a recent systematic review further indicates that associations between digital media use and ADHD symptoms may be reciprocal, with more consistent evidence for problematic digital media use than for screen time alone (5). At the same time, the broader literature on adolescent digital technology use has cautioned against treating “screen time” as a homogeneous exposure, because its associations with psychological outcomes are often small, heterogeneous, and dependent on how digital engagement is measured (6). Recent work on social media and adolescent mental health has also emphasized the need to move beyond total use and consider condition-specific and behavior-specific patterns of digital engagement (2). These findings support examining behavior-specific patterns of digital engagement in addition to broad measures of exposure duration. Accordingly, the present study investigated which specific digital behavior patterns were associated with different ADHD symptom dimensions, while recognizing that it did not directly compare these patterns with total screen time.

Among these patterns, fragmented information consumption may be particularly relevant. In the present study, fragmented information consumption refers to the tendency to obtain information through short, rapidly changing, and discontinuous content units, such as social media feeds, short-video platforms, and algorithmically curated streams. This pattern differs from general digital media use because it emphasizes discontinuity, rapid novelty, and limited sustained processing. Platform research suggests that content length and content presentation can shape user engagement and attention allocation (7), while emerging human-computer interaction research has begun to examine how short-form video consumption relates to sustained attention (8). For late adolescents and young adults, frequent fragmented information consumption may be statistically linked with inattention symptoms because it reflects a mode of engagement organized around rapid shifts of focus, immediate stimulation, and reduced tolerance for slower or more effortful tasks.

Media multitasking represents a second digital behavior pattern closely related to attention and self-regulation. It refers to engaging with multiple media streams or digital activities simultaneously, such as watching videos while messaging others, browsing social media while studying, or frequently switching between applications. Unlike fragmented information consumption, which emphasizes the discontinuous structure of content, media multitasking emphasizes concurrent or rapidly alternating digital activities. Prior research using network analytic approaches has shown that media multitasking and attention problems are closely interconnected rather than simply reflecting a single global media-use factor (9). This distinction is important because ADHD symptoms include both difficulty sustaining attention and difficulty regulating behavior in response to competing stimuli. Media multitasking may therefore serve as a behavioral context in which attentional instability and impulsive switching tendencies are more likely to be reported.

Speed watching is another emerging but less frequently examined digital behavior. It refers to watching video content at accelerated playback speed, often to increase efficiency or consume more information within a shorter period. Unlike media multitasking, speed watching does not necessarily involve multiple simultaneous tasks; instead, it increases the pace of information intake within a single media activity. Experimental research on lecture video speed suggests that moderate acceleration may have limited immediate costs under some learning conditions, whereas more extreme acceleration may impair comprehension (10). However, speed watching has rarely been examined in relation to ADHD symptom dimensions. Although the three behaviors may co-occur in everyday digital media use, they differ in their defining behavioral features and the attentional demands they may involve. Fragmented information consumption is defined primarily by the structure of the content being consumed: users encounter brief, heterogeneous, and weakly connected information units in sequence, which may repeatedly require attentional reorientation. Media multitasking is defined by the organization of attention across activities: users engage concurrently, or rapidly alternate, between multiple media streams or tasks, thereby dividing or switching attention across competing activities. Speed watching is defined by the temporal rate of information presentation: users accelerate a single, continuous media stream without necessarily introducing additional tasks or disrupting the continuity of the content. Thus, fragmented information consumption concerns the continuity and organization of information units, media multitasking concerns the number and coordination of competing streams or tasks, and speed watching concerns the processing rate of a single stream. These behaviors are therefore related but conceptually non-equivalent forms of digital engagement.

Although the present study focuses on digital behavior patterns, ADHD symptoms should not be interpreted outside broader developmental contexts. Environmental and family factors have both been discussed in relation to ADHD, although causal inference remains complex and many associations are heterogeneous (11, 12). Family environment is particularly relevant because parenting and parent-child interaction patterns are closely related to children’s and adolescents’ self-regulation, emotional development, and behavioral adjustment (12). Longitudinal evidence also suggests that parenting and child psychopathology symptoms may be bidirectionally and transactionally related over time (13). In contemporary families, parental phubbing may further represent a digitalized form of reduced parental responsiveness, as adolescents who perceive higher parental phubbing report lower connectedness with parents (14). In addition, perceived physical environments, including residential greenness, nature connectedness, and perceived pollution, may provide broader contextual information relevant to attention and regulation; for example, nature exposure has been discussed as potentially beneficial for attention-related difficulties in children with ADHD (15). In the present study, these family and perceived environmental factors are treated as ecological correlates rather than causal exposures. Conceptually, the present study adopts an ecologically informed multidomain perspective, drawing on the bioecological proposition that psychological functioning should be understood in relation to both individual characteristics and the contexts in which individuals participate (16). The framework is used here as an organizing heuristic rather than as a test of the complete bioecological process-person-context-time model. Specifically, age, gender, and subjective socioeconomic status represent person-level background characteristics; perceived greenness, nature connectedness, and pollution represent the perceived environmental domain; parental intrusion and parental phubbing represent the proximal family domain; and speed watching, fragmented information consumption, and media multitasking represent patterns of engagement with the contemporary digital environment. Considering these domains together allows the associations between digital behavior patterns and ADHD symptom dimensions to be examined beyond demographic, perceived environmental, and family correlates.

Despite growing interest in digital media and ADHD symptoms, several gaps remain. First, much of the existing literature has relied on broad indicators such as screen time, general internet use, or problematic digital media use, making it difficult to identify which specific digital behavior patterns are most relevant to ADHD symptom dimensions. Second, fragmented information consumption and speed watching remain underexamined, even though they are increasingly common in short-video and algorithm-driven media environments. Third, many studies focusing on digital behaviors have not simultaneously considered family and perceived environmental contexts, making it unclear whether specific digital behavior patterns explain additional variance in ADHD symptoms beyond broader ecological correlates.

To address these gaps, the present study examined the associations between three digital behavior patterns—fragmented information consumption, media multitasking, and speed watching—and two ADHD symptom dimensions, namely inattention and hyperactivity-impulsivity, among late adolescents and young adults. Guided by this ecologically informed multidomain framework, demographic characteristics were entered first as background covariates, followed by perceived environmental and family variables, whereas digital behavior variables were entered last because they constituted the focal predictors of the study. This block order was analytic rather than causal and allowed us to estimate the incremental variance associated with digital behavior patterns beyond the other measured domains. The conceptual organization of these domains is illustrated in **Figure 1**. Given the cross-sectional and self-report design, the study does not aim to infer causal effects. Instead, it seeks to provide a differentiated description of how specific digital behavior patterns are statistically associated with ADHD symptoms in late adolescents and young adults. Based on the preceding theoretical rationale, fragmented information consumption and media multitasking were expected to be positively associated with both inattention and hyperactivity-impulsivity symptoms, with fragmented information consumption expected to be particularly relevant to inattention. Because speed watching has rarely been examined in relation to ADHD symptom dimensions and the available evidence does not support a clear directional prediction, its associations were examined exploratorily. Accordingly, the study addressed the following research questions:

**Figure 1:**
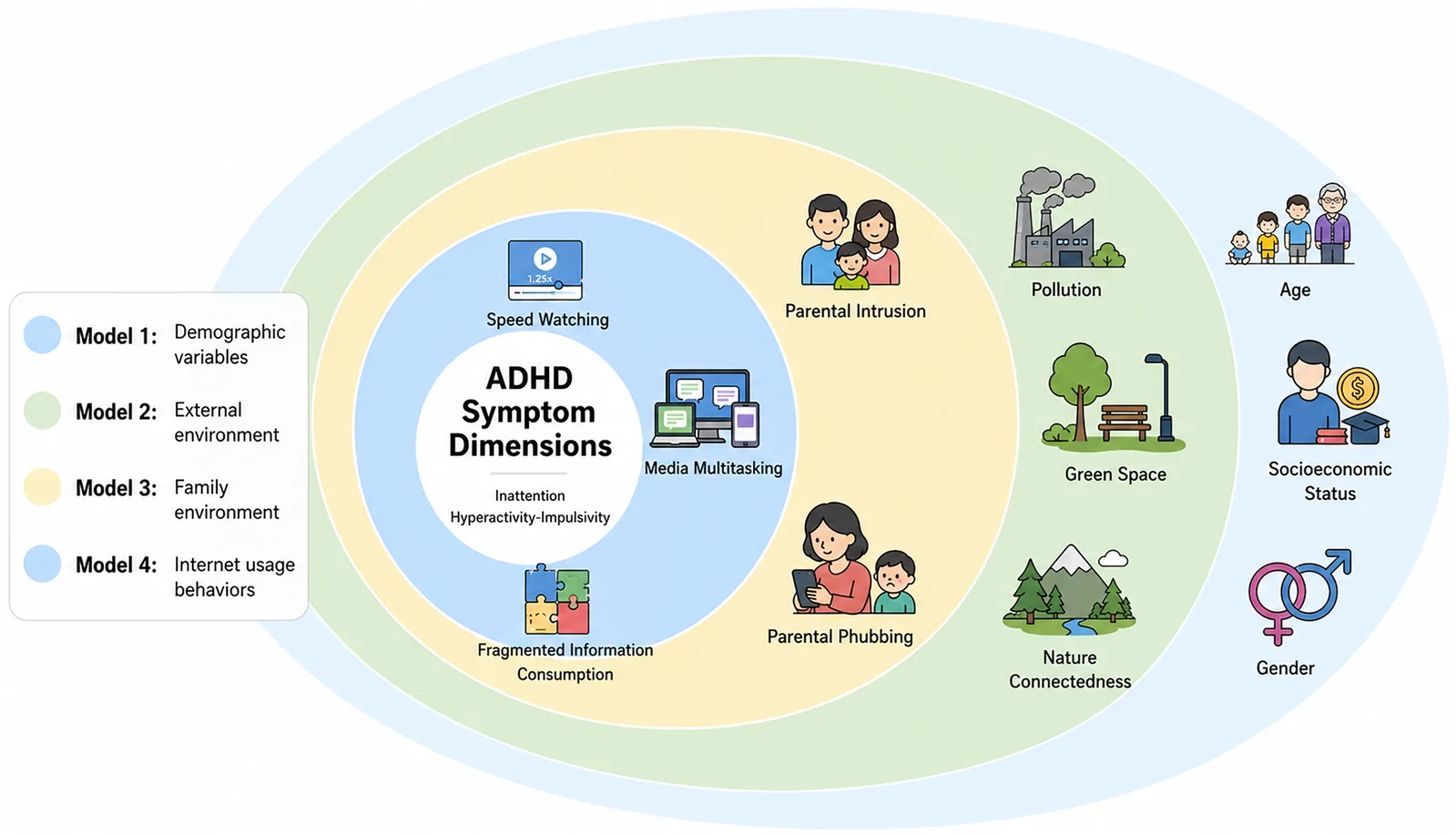
Ecologically informed multidomain conceptual framework of the study.

RQ1: What are the bivariate associations of fragmented information consumption, media multitasking, and speed watching with inattention and hyperactivity-impulsivity symptoms?
RQ2: When the three digital behaviors are considered simultaneously and demographic, perceived environmental, and family correlates are controlled, which digital behaviors retain unique associations with each ADHD symptom dimension?
RQ3: Does the digital behavior block explain incremental variance in inattention and hyperactivity-impulsivity symptoms beyond demographic, perceived environmental, and family correlates?

## 2 Methods

### 2.1 Participants

This cross-sectional study used convenience sampling to recruit 4,224 students from high schools and universities in Hunan, Shandong, and Guangdong provinces, China. After 806 responses were excluded based on the prespecified data-quality criteria, including an insufficient completion time of less than 240 seconds and other indicators of unreliable responding, the final analytic sample comprised 3,418 participants aged 15–25 years (M = 18.60, SD = 1.05). The sample included 1,929 high school students (56.44%) and 1,489 university students (43.56%), of whom 1,348 (39.44%) were male and 2,070 (60.56%) were female. A total of 396 participants (11.59%) were younger than 18 years. For the high school sample, class teachers assisted in distributing and collecting written informed consent forms signed by the students’ parents or legal guardians before the students completed paper-based questionnaires in person. The completed responses were subsequently entered into an electronic database. University students completed the questionnaire online through Wenjuanxing and provided electronic informed consent before accessing the survey. The study was conducted in accordance with the Declaration of Helsinki, and all recruitment, consent, and data-collection procedures were approved by the Medical Education Ethics Committee of Southern Medical University (SMUIRB-ME-202211003).

### 2.2 Measurement

Socioeconomic status was measured using a single item: “On a scale of 1-10, how do you perceive your socioeconomic status? (1 means you feel you belong to the lowest group, and 10 means you feel you belong to the highest group).” This item measures subjective perceptions of one’s economic status.

Green space and Pollution were measured using five items adapted from the Perceived Environmental questionnaire (17). Green space was assessed by two items: “The level of greenery in your community” and “The view of greenery from your home window,” with responses ranging from 1 (No greenery) to 5 (A lot of greenery). Pollution was measured by three items related to air, noise, and water pollution, such as: “How would you rate the air pollution in your living environment?” Responses were rated on a scale from 1 (Very dissatisfied) to 10 (Very satisfied). In this study, Cronbach’s alpha coefficients were 0.880 and 0.903, respectively.

Nature connectedness was assessed using the Inclusion of Nature in Self (INS) scale (18), which includes a single item: “Please choose the picture that best describes your relationship with the natural environment.” Responses ranged from 1 (where the circles touched but did not overlap) to 7 (where the circles were nearly entirely overlapping).

Parental intrusion was measured using four items from the “intrusion of child’s life and direction” factor of the Chinese Paternal/Maternal Overparenting Scale (CPOAS) (19). One example item is: “My parents interfere with my plans using their own agenda.” Responses ranged from 1 (Never) to 5 (Always). In this study, Cronbach’s alpha coefficient was 0.872.

Parental phubbing was assessed using eight items adapted from the Partner Phubbing scale (20). These eight items measured the frequency of phone use during interactions between the individual and their parents. An example item is: “My parents use their phones while we are eating together.” Responses ranged from 1 (Never) to 5 (Always). In this study, Cronbach’s alpha coefficient was 0.838.

Speed watching was measured by the Speed Watching scale using three items: “I often press and hold the screen to speed up playback,” “I prefer using the speed-up feature when watching videos,” and “I fast-forward through videos to save time.” These items measured the extent to which individuals used the fast-forward feature while watching videos. Responses ranged from 1 (Never) to 5 (Always). In this study, Cronbach’s alpha coefficient was 0.905. Psychometric tests of the scale and the original version are placed in **Supplementary Material 1**.

Fragmented information consumption was measured by the Fragmented Information Consumption scale using five items. These items assessed the degree to which individuals consumed fragmented information, with one example item being: “I watch short videos more than movies or TV shows.” Responses ranged from 1 (Never) to 5 (Always). In this study, Cronbach’s alpha coefficient was 0.849. Psychometric tests of the scale and the original version are placed in **Supplementary Material 2**.

Media multitasking was assessed using four items from the study by Zhong et al. (21). These items measured the extent to which individuals engaged in media multitasking or used multiple electronic devices simultaneously. An example item is: “I often find myself using several media technological devices simultaneously.” Responses ranged from 1 (Never) to 5 (Always). In this study, Cronbach’s alpha coefficient was 0.844. Although these digital behaviors may overlap in practice, the three measures target different defining features: discontinuity across information units for fragmented information consumption, engagement with multiple competing streams or tasks for media multitasking, and accelerated presentation within a single continuous stream for speed watching.

ADHD was measured using the Chinese Short Version of the Adult ADHD Self-Report Scale (ASRS-CSV), which has been demonstrated to be an effective and reliable tool for assessing adult ADHD symptoms (22), and widely used among Chinese teenagers (23, 24). The scale includes two factors: Inattention and Hyperactivity-Impulsivity, each comprising three items. Example items for these factors include: “I procrastinate or avoid dealing with tough tasks” (Inattention) and “I feel restless or irritable” (Hyperactivity-Impulsivity). Responses ranged from 1 (Never) to 5 (Always). In this study, the Cronbach’s alpha coefficients for the total scale and the two subscales were 0.903, 0.874, and 0.838, respectively. Additionally, the applicability of the scale to adolescents was validated in **Supplementary Material 3** of this study.

### 2.3 Data analysis

All statistical analysis was performed using SPSS 26.0 and R Statistical Software (25). Before conducting the analysis, missing values and outliers were examined. Cronbach’s alpha was conducted using SPSS 26.0. Descriptive statistical analyses were then performed on variables. Using R Statistical Software, Pearson correlation analysis was employed to explore the relationships between variables. The hierarchical regression analysis was performed, with models with inattention symptoms and hyperactivity-impulsivity symptoms as outcome variables, progressively incorporating different predictors. Model 1 included demographic variables (age, gender, socioeconomic status); Model 2 added environmental variables (GS, NC, P); Model 3 incorporated family variables (PI, PP); and Model 4 further included digital behavior variables (SW, FIC, MM). The block-entry order was not preregistered or specified in a formal analysis protocol; however, it was determined on conceptual grounds before inspection of the hierarchical regression results. The sequence followed the conceptual organization described above: demographic characteristics were treated as baseline covariates, perceived environmental and family variables represented contextual domains, and digital behavior variables were entered last because they constituted the focal domain of the study. Entering the digital behavior variables at the final step was intended to evaluate their incremental explanatory contribution after accounting for the preceding variables and did not imply temporal, causal, or inherent explanatory priority. Because different predictor domains may share explanatory variance and changes in R² depend on the order of block entry, block-specific changes in R² were interpreted as incremental variance conditional on the specified sequence rather than as evidence of the absolute or relative importance of the different domains. Multicollinearity was assessed for each hierarchical regression model using variance inflation factors (VIFs) and tolerance values. VIF values below 5 and tolerance values above 0.20 were considered indicative of acceptable levels of multicollinearity. Pearson correlation analyses were used to address RQ1. The coefficients of the three digital behavior variables in the fully adjusted models were used to address RQ2, while the change in R² associated with the addition of the digital behavior block was used to address RQ3.

## 3 Results

### 3.1 Descriptive statistical analysis

**Table 1** shows the results of descriptive statistical analysis of variables. The final sample comprised 3,418 participants aged 15–25 years, including 1,929 high school students (56.44%) and 1,489 university students (43.56%). Of these participants, 1,348 (39.44%) were male and 2,070 (60.56%) were female. The mean age was 18.60 years (SD = 1.05), and the mean subjective socioeconomic status score was 3.93 (SD = 1.86). The mean inattention and hyperactivity-impulsivity symptom scores were 8.29 (SD = 2.37) and 7.79 (SD = 2.39), respectively.

**Table 1:** Descriptive statistics (*n* = 3418).

Variable	*M*/*n*	*SD*/%
Age	18.60	1.05
Gender:		
Male	1348	39.44
Female	2070	60.56
SS	3.93	1.86
GS	7.64	1.84
NC	4.16	1.62
P	11.92	5.21
PI	9.32	3.31
PP	20.74	5.51
FIC	14.22	3.77
SW	9.27	3.03
MM	11.61	3.05
Inattention symptoms	8.29	2.37
Hyperactivity-impulsivity symptoms	7.79	2.39

SS, socioeconomic status; GS, green space; NC, nature connectedness; P, pollution; PI, parental intrusion; PP, parental phubbing; SW, speed watching; FIC, fragmented information consumption; MM, media multitasking.

### 3.2 Pearson correlation analysis (RQ1)

The results of the Pearson correlation analysis are presented in **Figure 2**. The correlation coefficients between each predictor variable and inattention symptoms were all significant. Similarly, the correlation coefficients between each predictor variable and hyperactivity-impulsivity symptoms were also significant, except for age and gender. Among these predictor variables, FIC had the highest correlation coefficient with inattention symptoms (0.588) and also with hyperactivity-impulsivity symptoms (0.488). The two symptom dimensions, inattention and hyperactivity-impulsivity, were also substantially correlated (r = 0.733).

**Figure 2:**
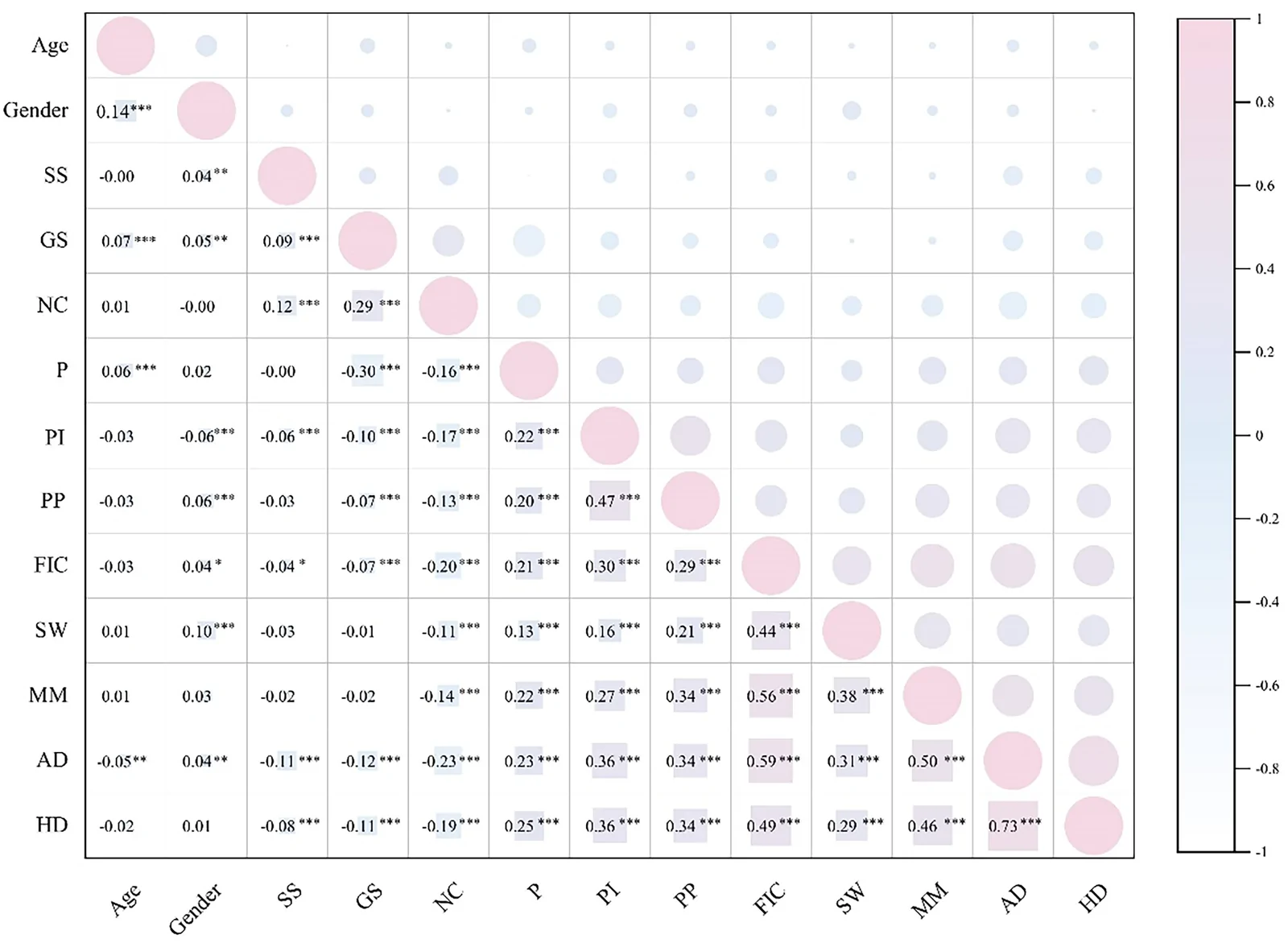
Correlation heatmap. *p < 0.05; **p < 0.01; ***p < 0.001.

### 3.3 Hierarchical regression analysis (RQs 2 and 3)

The hierarchical regression results of various predictor variables for inattention and hyperactivity-impulsivity symptoms are presented in **Table 2**. This study employed four models incorporating different predictor variables to construct hierarchical regression models with inattention and hyperactivity-impulsivity symptoms as the outcome variables, respectively. No evidence of problematic multicollinearity was observed. Across Models 1–4, VIF values ranged from 1.002 to 1.689. In the fully adjusted Model 4, VIF values ranged from 1.023 to 1.689 (mean VIF = 1.275), and tolerance values ranged from 0.592 to 0.978. Because the same set of predictors was used in the inattention and hyperactivity-impulsivity models, the multicollinearity diagnostics were identical for the two outcomes. Although the two symptom dimensions were correlated, they were analyzed as separate dependent variables and neither was entered as a predictor of the other; therefore, their correlation did not contribute to multicollinearity in either regression model. In the case of inattention symptoms as the outcome variable, Model 1 explained 1.8% of the variance, with socioeconomic status being the strongest predictor (*β* = -0.116, *p* < 0.001). Compared to Model 1, Model 2 accounted for an additional 8.9% of the variance, with P being the strongest predictor (*β* = 0.207, *p* < 0.001). Model 3 explained 11.0% more variance than Model 2, with PI as the strongest predictor (*β* = 0.220, *p* < 0.001). When the digital behavior variables were entered in Model 4, the model accounted for an additional 22.3% of the variance in inattention beyond the variables entered in Models 1–3 (ΔR² = 0.223). Within Model 4, FIC showed the largest standardized regression coefficient (*β* = 0.379, *p* < 0.001). In the final model, all predictor variables were significant except for SW. In the case of HD as the outcome variable, Model 1 explained 0.7% of the variance, with socioeconomic status being the strongest predictor (*β* = -0.082, *p* < 0.001). Model 2 accounted for an additional 8.5% of the variance compared to Model 1, with P being the strongest predictor (*β* = 0.237, *p* < 0.001). Model 3 explained 11.2% more variance than Model 2, with PI as the strongest predictor (*β* = 0.217, *p* < 0.001). When the digital behavior variables were entered in Model 4, the model accounted for an additional 14.5% of the variance in hyperactivity-impulsivity beyond the variables entered in Models 1–3 (ΔR² = 0.145). Within Model 4, FIC showed the largest standardized regression coefficient (*β* = 0.256, *p* < 0.001). In the final model, all predictor variables were significant except for age, gender, and GS. When entered at the final step, the digital behavior block accounted for substantial additional variance beyond the demographic, perceived environmental, and family variables entered in the preceding models: 22.3% for inattention and 14.5% for hyperactivity-impulsivity. Because changes in R² are conditional on the order in which predictor blocks are entered, these results should not be interpreted as demonstrating that the digital behavior domain had greater explanatory power or importance than the preceding domains.

**Table 2:** Hierarchical regression analysis.

Predictor	Inattention symptoms (*β*)				Hyperactivity-impulsivity symptoms (*β*)			
variable	Model 1	Model 2	Model 3	Model 4	Model 1	Model 2	Model 3	Model 4
Age	-0.054**	-0.063***	-0.047**	-0.038**	-0.026	-0.037*	-0.022	-0.016
Gender (Female)	0.057***	0.052**	0.055***	0.035**	0.012	0.007	0.009	-0.009
SS	-0.116***	-0.094***	-0.081***	-0.074***	-0.082***	-0.066***	-0.053***	-0.048***
GS		0.006	0.001	-0.029*		0.012	0.007	-0.021
NC		-0.186***	-0.140***	-0.068***		-0.143***	-0.096***	-0.039**
P		0.207***	0.127***	0.047***		0.237***	0.156***	0.089***
PI			0.220***	0.129***			0.217***	0.145***
PP			0.184***	0.071***			0.191***	0.094***
SW				0.005				0.038*
FIC				0.379***				0.256***
MM				0.206***				0.203***
*R* ^2^	0.018	0.107	0.217	0.440	0.007	0.092	0.204	0.349
Adjusted *R*^2^	0.017	0.105	0.215	0.438	0.006	0.090	0.203	0.347
Δ*R*^2^	–	0.089	0.110	0.223	–	0.085	0.112	0.145

*p < 0.05. **p < 0.01. ***p < 0.001. SS, socioeconomic status; GS, green space; NC, nature connectedness; P, pollution; PI, parental intrusion; PP, parental phubbing; SW, speed watching; FIC, fragmented information consumption; MM, media multitasking.

## 4 Discussion

The present study examined three specific digital behavior patterns in relation to two ADHD symptom dimensions within an ecologically informed multidomain framework. Regarding RQ1, all three digital behaviors showed positive bivariate associations with both inattention and hyperactivity-impulsivity symptoms, although the magnitudes differed across behaviors and symptom dimensions. Regarding RQ2, fragmented information consumption and media multitasking retained unique positive associations with both symptom dimensions after adjustment for demographic, perceived environmental, family, and other digital behavior variables. Speed watching, by contrast, was not uniquely associated with inattention and showed only a small positive association with hyperactivity-impulsivity. Regarding RQ3, the digital behavior block accounted for an additional 22.3% of the variance in inattention and 14.5% of the variance in hyperactivity-impulsivity beyond the preceding blocks. These increments should be interpreted as conditional on the specified block-entry sequence. Because the predictor domains may share explanatory variance, a different block-entry order could yield different block-specific changes in R²; therefore, the observed ΔR² values do not indicate that digital behaviors are inherently more important than the demographic, perceived environmental, or family domains. Taken together, these findings indicate that the three digital behaviors were not uniformly associated with ADHD symptoms and underscore the value of distinguishing among behavior-specific forms of digital engagement. Because aggregate measures such as total screen time were not included, no conclusions can be drawn regarding their relative or incremental explanatory value.

The strongest finding concerned fragmented information consumption. This behavior remained the most prominent correlate of both inattention and hyperactivity-impulsivity after demographic, perceived environmental, and family factors had been considered. This result is theoretically meaningful because fragmented information consumption captures a mode of digital engagement characterized by short, rapidly changing, discontinuous, and novelty-rich content. It therefore differs from general screen time, which only measures duration of exposure, and from general internet use, which does not specify how information is processed. Recent research has increasingly questioned the explanatory value of broad screen-time indicators and has emphasized the need to examine specific digital behaviors and platform-specific usage patterns (6, 26). In this sense, the present findings support a shift from asking whether digital media use is associated with ADHD symptoms to asking which forms of digital engagement are most strongly linked to attention- and impulse-related symptom dimensions.

The association between fragmented information consumption and inattention is especially consistent with emerging evidence on short-form and rapidly changing digital content. Short-form videos and algorithmically curated feeds are designed around quick transitions, immediate novelty, and highly salient stimuli. Recent studies have suggested that short-form video media use is associated with poorer sustained attention and greater inattentive behaviors, even when other factors such as total screen time are considered (8, 27). Although fragmented information consumption in the present study is broader than short-form video use alone, these findings converge in suggesting that digital environments organized around rapid attentional shifts may be particularly relevant to inattention. The present result extends this literature by showing that fragmented information consumption was not only associated with inattention but also with hyperactivity-impulsivity in a large sample of late adolescents and young adults. The prominence of fragmented information consumption may be interpreted in relation to the interaction between platform affordances, habit formation, and user selection. Algorithmically refreshed feeds and low-friction interactions, such as continuous swiping and autoplay, repeatedly present brief and novel content with few natural stopping points. These affordances may support short cycles of engagement in which attention is repeatedly redirected toward newly presented stimuli, and repeated engagement may gradually become habitual (7, 8). The reverse pathway is also plausible: individuals with higher inattention or hyperactivity-impulsivity symptoms may be particularly attracted to environments that provide frequent novelty and stimulation while placing fewer demands on prolonged engagement with a single information source. The observed association may therefore reflect platform-supported patterns of habitual engagement, symptom-related selection of stimulating content, or reciprocal reinforcement between these processes.

Media multitasking also showed robust associations with both ADHD symptom dimensions. This result is consistent with the theoretical expectation that simultaneous or rapidly alternating engagement with multiple media streams may be closely related to attentional instability, distractibility, and self-regulation difficulties. Unlike fragmented information consumption, which emphasizes the discontinuous structure of information, media multitasking emphasizes concurrent or rapidly shifting digital activities. Prior work using network analytic approaches has shown that media multitasking and attention problems are closely interconnected and that different forms of media multitasking may not be reducible to a single global media-use construct (9). The present finding that media multitasking remained associated with ADHD symptoms after fragmented information consumption and speed watching were included suggests that it captures a distinct aspect of digital behavior. This supports the value of separating digital behavior patterns rather than treating them as interchangeable indicators of general digital media exposure. Media multitasking may similarly be understood in relation to both digital affordances and stimulation-seeking tendencies. Notifications, rapid switching between applications, and interfaces that allow several streams to remain active simultaneously make it easy to alternate repeatedly between competing activities. These features may facilitate habitual task switching and increase the frequency with which attention is redirected before an ongoing task is completed. Conversely, individuals with greater attentional instability, restlessness, or impulsivity may actively maintain several streams because multiple sources of input provide greater stimulation or relief from tasks experienced as slow or insufficiently engaging. Repeated selection of multitasking environments and repeated switching between activities may therefore reinforce one another over time, although the present cross-sectional data cannot determine the direction of this relationship (4, 5, 9).

Speed watching showed a more limited association pattern. In the final models, speed watching was not significantly associated with inattention but showed a small positive association with hyperactivity-impulsivity. This weaker pattern may reflect the fact that speed watching differs structurally from both fragmented information consumption and media multitasking. Speed watching increases the rate of information intake within a single media activity, but it does not necessarily involve discontinuous content, algorithmically shifting topics, or competing media streams. Experimental work on accelerated video viewing suggests that moderate playback speed may not necessarily impair immediate learning under all conditions, although higher levels of acceleration may reduce comprehension (10). Although playback-speed control is also a digital platform affordance, it primarily changes the rate at which a single, continuous stream is presented rather than introducing additional content streams or repeated shifts between competing goals. Speed watching may therefore represent an intentional efficiency or engagement strategy in some users rather than a habitual pattern of attentional switching. At the same time, its small association with hyperactivity-impulsivity may partly reflect stimulation-seeking preferences or reduced tolerance for slowly presented information. These characteristics may help explain why speed watching showed a less consistent association pattern than fragmented information consumption and media multitasking, although this interpretation requires direct testing.

The more consistent associations observed for fragmented information consumption and media multitasking may be understood in terms of the attentional demands that distinguish them from speed watching. Fragmented information consumption involves sequential exposure to brief, heterogeneous, and weakly connected information units. Each transition introduces a new attentional target and may require repeated disengagement, reorientation, and reconstruction of the immediate processing goal. Media multitasking, by contrast, involves concurrent or rapidly alternating engagement with multiple streams or tasks and may therefore place demands on maintaining competing goals, inhibiting irrelevant information, and switching attention between activities (9). Both behaviors involve repeated changes or competition among attentional targets, characteristics that are behaviorally relevant to difficulties in sustained attention and self-regulation.

Speed watching increases the rate of information intake but generally preserves a single attentional target, a continuous content structure, and a stable task goal. It may also be used deliberately as an efficiency or engagement strategy rather than necessarily reflecting poorly regulated digital behavior. Experimental evidence suggests that moderate playback acceleration may have limited immediate cognitive costs under some conditions, whereas less favorable effects may emerge at higher speeds (10). In addition, the present speed-watching measure assessed general frequency or preference but did not distinguish between moderate and extreme playback speeds or between different types of video content. This heterogeneity may partly explain why speed watching showed no unique association with inattention and only a small association with hyperactivity-impulsivity after fragmented information consumption and media multitasking were included. The stronger patterns observed for the latter two behaviors may also partly reflect their greater conceptual proximity to distractibility, switching, and competition among activities. These explanations remain tentative because attentional reorientation, task switching, inhibitory control, processing load, and actual digital behavior were not measured directly.

Importantly, the observed associations are compatible with two plausible directions, neither of which can be prioritized on the basis of the present cross-sectional data. Repeated engagement with rapidly changing, discontinuous, or competing digital stimuli may contribute to or reinforce difficulties in sustained attention and behavioral regulation. Conversely, individuals with higher pre-existing ADHD symptoms may selectively prefer these forms of digital engagement. Those with greater inattention symptoms may find brief and rapidly changing content easier to remain engaged with, whereas those with greater hyperactivity-impulsivity symptoms may be more inclined to seek novelty, faster stimulation, frequent switching, or simultaneous activities. A reciprocal process is also possible, in which symptom-related preferences shape digital behavior and repeated engagement with these environments subsequently reinforces the same behavioral tendencies (3–5).

Previous research has suggested that behaviorally dysregulated forms of digital engagement may provide information that is not captured by exposure duration alone. For example, a longitudinal study found that addictive-use trajectories of social media, mobile phones, and video games were associated with poorer mental health outcomes, whereas baseline total screen time was not associated with those outcomes after adjustment (26). However, that study examined broader mental health outcomes, and the present study did not assess total screen time. The current findings therefore do not demonstrate that fragmented information consumption or media multitasking is more informative than screen duration. Instead, they provide behavior-specific information that future studies should examine alongside measures of total screen exposure.

Although digital behavior patterns were the central focus, the findings across the contextual blocks are better understood as components of an interconnected ecological context rather than as isolated predictors. Subjective socioeconomic status reflects the broader resource context, perceived nature connectedness and pollution capture aspects of participants’ experienced physical environment, and parental intrusion and parental phubbing represent more proximal relational conditions. Lower subjective socioeconomic status, lower nature connectedness, greater perceived pollution, and higher parental intrusion and parental phubbing were generally associated with higher ADHD symptom scores. Viewed together, these patterns suggest that ADHD symptoms and digital engagement occur within broader material, environmental, and family contexts that may support or constrain attention and self-regulation.

These contextual domains may also be related to digital engagement rather than operating independently from it. Broader resource constraints and less restorative environmental experiences may coincide with stress and fewer opportunities for sustained attention, whereas parental intrusion and parental phubbing may be linked to autonomy, parental responsiveness, and family routines surrounding device use (11–14). At the same time, ADHD symptoms may shape young people’s perceptions of their environments, their digital choices, and the ways in which family members respond to them. The attenuation of the parental coefficients after the digital behavior variables were entered is therefore consistent with shared variance across the family and digital domains, but it does not establish that digital behaviors mediate the associations between family factors and ADHD symptoms. Because the measures were cross-sectional and self-reported—and the environmental variables reflected perceived rather than objectively assessed conditions—the temporal order and mechanisms connecting these domains cannot be determined. Longitudinal studies that jointly assess family processes, objective and perceived environmental conditions, and digital behavior are needed to examine these interrelationships.

Taken together, the main theoretical contribution of this study lies in differentiating among forms of digital engagement with distinct attentional characteristics rather than treating digital-media use as a single, homogeneous construct. Fragmented information consumption, characterized by discontinuity and rapid novelty, and media multitasking, characterized by competition and switching across multiple streams, showed stronger and more consistent associations with both ADHD symptom dimensions. By contrast, speed watching, which increases the presentation rate while generally preserving a single continuous stream, was not uniquely associated with inattention and showed only a small association with hyperactivity-impulsivity. These differential patterns indicate that the three behaviors should not be treated as interchangeable indicators of digital-media use. Instead, theoretical models should specify which structural characteristics of digital engagement—such as content discontinuity, competition among attentional targets, or temporal acceleration—are hypothesized to relate to particular ADHD symptom dimensions. These findings concern differences in patterns of association and do not establish causal effects or demonstrate that behavior-specific measures are more informative than total screen time, which was not assessed. Longitudinal research incorporating both duration-based and behavior-specific measures is needed to examine reciprocal relationships and determine whether these distinctions provide incremental explanatory value.

## 5 Implication

These findings may inform future educational guidance, family communication, and mental health promotion among late adolescents and young adults. Digital media guidance could consider not only the duration of screen exposure but also how young people engage with digital content, including fragmented information consumption, habitual app switching, and media multitasking during learning or work. Because total screen time was not assessed, however, the present study cannot determine the relative importance of exposure duration and specific engagement patterns. Educators and families may find it useful to distinguish among different forms of digital engagement rather than treating all digital behaviors as equivalent, as fragmented information consumption and media multitasking showed stronger and more consistent statistical associations with ADHD symptom dimensions than speed watching. These behaviors should be regarded as potential behavioral correlates or markers of attentional and self-regulatory difficulties rather than as established modifiable causes. Intentional digital routines, fewer competing digital activities during academic work, and structured periods of single-task engagement may be considered as optional attention-supportive approaches. However, the effectiveness of these approaches in preventing or reducing ADHD symptoms was not tested in the present study and requires evaluation in longitudinal and intervention research. Any guidance should therefore be individualized, should avoid attributing ADHD symptoms solely to digital media behavior, and should not imply that changing these behaviors will necessarily improve ADHD symptoms.

## 6 Limitations and future directions

Several limitations should be acknowledged. First, the cross-sectional design prevents causal inference. The observed associations may indicate that specific digital behavior patterns are related to ADHD symptoms, but they cannot determine whether digital behaviors contribute to symptoms, whether individuals with higher ADHD symptoms are more likely to engage in fragmented and multitasking-oriented digital behaviors, or whether both are explained by third variables. Longitudinal studies with repeated assessments of both ADHD symptoms and digital behaviors are needed to examine temporal precedence and reciprocal associations. Cross-lagged or intensive longitudinal designs combining self-reports with digital trace data or ecological momentary assessment would be particularly useful for determining whether within-person changes in symptoms precede changes in digital behavior, whether the reverse occurs, or whether both processes operate over time. Second, all variables were assessed by self-report, which may increase common method bias and inflate associations among digital behavior patterns and ADHD symptoms. In addition, the proposed explanations involving attentional reorientation, task switching, inhibitory control, and processing rate were not tested directly. Future studies should combine objective digital trace measures with behavioral assessments of sustained attention and executive control to examine whether these processes account for the different association patterns across digital behaviors. Future research should incorporate objective digital trace data, app-use logs, ecological momentary assessment, informant reports, and behavioral attention tasks. Third, the sample was non-clinical, and ADHD symptoms were measured dimensionally rather than through clinical diagnosis. The findings should therefore be interpreted as evidence regarding self-reported ADHD symptom variation rather than clinically diagnosed ADHD. Fourth, several important covariates were not included, such as sleep quality, depression, anxiety, academic stress, total screen time, problematic smartphone use, and objectively measured digital exposure. Consequently, the present study cannot determine whether fragmented information consumption, media multitasking, or speed watching account for incremental variance in ADHD symptoms beyond total screen time, nor can it establish that these behavior-specific indicators are more informative than exposure duration. Future studies should include duration-based and behavior-specific measures simultaneously. Including these variables would help clarify whether fragmented information consumption and media multitasking remain robustly associated with ADHD symptoms beyond broader mental health and lifestyle factors. Finally, the perceived environmental variables used in this study should not be treated as objective environmental exposure indicators. Future research could combine perceived environmental measures with geospatial, sensor-based, or administrative environmental data.

## 7 Conclusion

This study found that specific digital behavior patterns, particularly fragmented information consumption and media multitasking, were strongly associated with self-reported ADHD symptoms in late adolescents and young adults. Fragmented information consumption was the most prominent correlate of both inattention and hyperactivity-impulsivity, while speed watching showed a weaker and more limited association. These findings suggest that research on digital media and ADHD symptoms may benefit from assessing specific forms of digital engagement alongside general measures of screen exposure. Studies that include both types of measures are needed to determine their relative and incremental explanatory value. These associations may reflect the influence of digital engagement on attentional functioning, the selection of particular digital environments by individuals with higher pre-existing symptoms, reciprocal reinforcement between the two, or shared underlying factors. Given the cross-sectional and self-report design, the findings should be interpreted as statistical associations rather than causal effects.

## Data Availability

The raw data supporting the conclusions of this article will be made available by the authors, without undue reservation.
