# Supplementary material for: Digital behavior patterns and ADHD symptoms in late adolescents and young adults
Source: Front Psychiatry. 2026 Oct 8;17:1968257. doi: 10.3389/fpsyt.2026.1968257 (PMC13687006; doi:10.3389/fpsyt.2026.1968257)
Supplement: Supplementary file 1 [file SupplementaryFile1.pdf]

Supplementary Material 1

Psychometric properties and item content of the Speed Watching Scale

Three-item scale used in the present study

Purpose and measure

This supplement reports the item wording, scoring, internal consistency, and factor-analytic results for the Speed Watching Scale.

Scoring. Each item was rated from 1 (Never) to 5 (Always). The three responses were summed, yielding a possible total score from 3 to 15; higher scores indicate more frequent speed-watching behavior.

Item content and item-level results

Table S1. Speed Watching Scale item wording and psychometric statistics

Item	Item wording	Mean	SD	Corrected item-total r	Std. loading
1	I often press and hold the screen to speed up playback.	3.20	1.08	0.839	0.933
2	I prefer using the speed-up feature when watching videos.	3.09	1.13	0.869	0.981
3	I fast-forward through videos to save time.	2.98	1.09	0.730	0.785

Note. N = 3,418. All loadings were statistically different from zero, $p < .001$.

Psychometric analysis

Analyses used the cleaned study sample. Cronbach's alpha was calculated from raw item scores. Because the items used five ordered response categories, confirmatory factor analysis used the robust weighted least squares estimator (WLSMV). Standardized loadings are reported. Composite reliability (CR) and average variance extracted (AVE) were calculated from the standardized loadings and residual variances.

Table S2. Scale-level reliability and convergent validity

N	Observed mean	Observed SD	Cronbach's alpha	CR	AVE
3418	9.27	3.03	0.905	0.930	0.817

The one-factor CFA with three indicators was just identified (zero degrees of freedom), so global fit indices are not informative. The standardized loadings ranged from .785 to .981. Together with alpha = .905, CR = .930, and AVE = .817, the results indicate strong internal consistency and convergent measurement evidence in the study sample.

Software and reproducibility

Data screening, descriptive statistics, and Cronbach's alpha were completed in IBM SPSS Statistics 26.0. Supplementary CFA, CR, and AVE analyses were conducted in R 4.5.0 with lavaan 0.6-20 using WLSMV estimation. No responses were missing for the analyzed items.
