# Supplementary material for: Digital behavior patterns and ADHD symptoms in late adolescents and young adults
Source: Front Psychiatry. 2026 Oct 8;17:1968257. doi: 10.3389/fpsyt.2026.1968257 (PMC13687006; doi:10.3389/fpsyt.2026.1968257)
Supplement: Supplementary file 2 [file SupplementaryFile2.pdf]

Supplementary Material 2

Psychometric properties and item content of the Fragmented Information Consumption Scale

Five-item scale used in the present study

Purpose and measure

This supplement reports the item wording, scoring, internal consistency, and factor-analytic results for the Fragmented Information Consumption Scale. The scale assesses disconnected, aimless, and rapidly shifting patterns of online information consumption.

Scoring. Each item was rated from 1 (Never) to 5 (Always). The five responses were summed, yielding a possible total score from 5 to 25; higher scores indicate more frequent fragmented information consumption.

Item content and item-level results

Table S1. Fragmented Information Consumption Scale item wording and psychometric statistics

Item	Item wording	Mean	SD	Corrected item-total r	Std. loading
ISB1	I watch short videos more than movies or TV shows.	3.09	0.95	0.509	0.582
ISB2	I often lose track of my original purpose when searching for information online.	2.79	0.96	0.679	0.772
ISB3	Overall, the content I watch online is diverse but lacks connection.	2.85	0.95	0.722	0.836
ISB4	I watch more lighthearted or nonsensical information rather than logically structured content.	2.76	0.92	0.719	0.839
ISB5	I often switch between several apps, browsing aimlessly.	2.73	1.00	0.674	0.783

Note. N = 3,418. All loadings were statistically different from zero, $p < .001$.

Psychometric analysis

Analyses used the cleaned study sample. Cronbach's alpha was calculated from raw item scores. Because the items used five ordered response categories, confirmatory factor analysis used the robust weighted least squares estimator (WLSMV). Standardized loadings are reported. Composite reliability (CR) and average variance extracted (AVE) were calculated from the standardized loadings and residual variances.

Table S2. Scale-level reliability and convergent validity

N	Observed mean	Observed SD	Cronbach's alpha	CR	AVE
3418	14.22	3.77	0.849	0.876	0.590

Table S3. One-factor confirmatory factor analysis

Scaled chi-square (df)	p	CFI	TLI	RMSEA	90% CI	SRMR
209.42 (5)	< .001	0.991	0.981	0.109	0.097-0.122	0.021

The standardized loadings ranged from .582 to .839. CFI, TLI, and SRMR supported the one-factor representation, whereas RMSEA indicated residual misfit. The latter should be interpreted with care because the model had only five

degrees of freedom. Overall reliability and convergent evidence were acceptable (alpha = .849, CR = .876, AVE = .590), while the global fit results suggest that the single-factor structure should be reassessed in an independent sample.

Software and reproducibility

Data screening, descriptive statistics, and Cronbach's alpha were completed in IBM SPSS Statistics 26.0. Supplementary CFA, CR, and AVE analyses were conducted in R 4.5.0 with lavaan 0.6-20 using WLSMV estimation. No responses were missing for the analyzed items.
