# Supplementary material for: Digital behavior patterns and ADHD symptoms in late adolescents and young adults
Source: Front Psychiatry. 2026 Oct 8;17:1968257. doi: 10.3389/fpsyt.2026.1968257 (PMC13687006; doi:10.3389/fpsyt.2026.1968257)
Supplement: Supplementary file 3 [file SupplementaryFile3.pdf]

Supplementary Material 3

Adolescent validation of the ASRS-CSV

Reliability and factor structure in participants aged 15 to 19 years

Purpose and measure

This supplement evaluates the Chinese Short Version of the Adult ADHD Self-Report Scale (ASRS-CSV) in the adolescent subset of the study sample. The six-item measure contains three Inattention items and three Hyperactivity-Impulsivity items.

Scoring. Each item was rated from 1 (Never) to 5 (Always). Subscale scores range from 3 to 15, and the total score ranges from 6 to 30. Higher scores indicate more frequent ADHD-related symptoms. The measure was analyzed as a research screening instrument and was not used to establish a clinical diagnosis.

Item content

Table S1. ASRS-CSV item wording and subscale assignment

Item	Subscale	Item wording
AD1	Inattention	I procrastinate or avoid dealing with tough tasks.
AD2	Inattention	I often misplace or lose things in daily life.
AD3	Inattention	I get distracted by irrelevant things.
HD1	Hyperactivity-Impulsivity	I feel restless or irritable.
HD2	Hyperactivity-Impulsivity	I feel tense and find it hard to relax.
HD3	Hyperactivity-Impulsivity	I feel overly hyperactive, as if "on edge."

Samples and analysis

The full cleaned sample contained 3,418 participants. The adolescent validation subset included 2,768 participants aged 15 to 19 years (M = 18.16, SD = 0.66); 1,179 (42.6%) were male and 1,589 (57.4%) were female. There were no missing ASRS-CSV item responses in either sample.

Analyses used the cleaned study sample. Cronbach's alpha was calculated from raw item scores. Because the items used five ordered response categories, confirmatory factor analysis used the robust weighted least squares estimator (WLSMV). Standardized loadings are reported. Composite reliability (CR) and average variance extracted (AVE) were calculated from the standardized loadings and residual variances.

A correlated two-factor model representing Inattention and Hyperactivity-Impulsivity was tested in the adolescent subset. A one-factor model was also estimated as a structural comparison.

Reliability

Table S2. ASRS-CSV score distributions and internal consistency

Sample	Score	N	Mean	SD	Cronbach's alpha
Full sample	Total	3418	16.08	4.43	0.903
Full sample	Inattention	3418	8.29	2.37	0.874

Sample	Score	N	Mean	SD	Cronbach's alpha
Full sample	Hyperactivity-Impulsivity	3418	7.79	2.39	0.838
Age 15-19	Total	2768	16.14	4.35	0.896
Age 15-19	Inattention	2768	8.34	2.34	0.868
Age 15-19	Hyperactivity-Impulsivity	2768	7.80	2.36	0.827

The full-sample coefficients reproduce the values reported in the main article after rounding: total alpha = .903, Inattention alpha = .874, and Hyperactivity-Impulsivity alpha = .838. Internal consistency remained high in the adolescent subset.

Factorial and convergent validity in adolescents

Table S3. Confirmatory factor analysis in participants aged 15 to 19 years

Model	Scaled chi-square (df)	CFI	TLI	RMSEA	90% CI	SRMR
Correlated two-factor	150.29 (8)	0.997	0.995	0.080	0.069-0.092	0.016
One-factor	1970.42 (9)	0.960	0.933	0.281	0.270-0.291	0.056

Note. For both models, $p < .001$ for the scaled chi-square test.

Table S4. Adolescent item statistics and standardized two-factor loadings

Item	Factor	Mean	SD	Std. loading
AD1	Inattention	2.70	0.89	0.832
AD2	Inattention	2.83	0.89	0.837
AD3	Inattention	2.76	0.87	0.926
HD1	Hyperactivity-Impulsivity	2.67	0.93	0.945
HD2	Hyperactivity-Impulsivity	2.59	0.92	0.924
HD3	Hyperactivity-Impulsivity	2.53	0.90	0.631

Note. N = 2,768. All loadings were statistically different from zero, $p < .001$.

Table S5. Adolescent composite reliability and average variance extracted

Factor	CR	AVE
Inattention	0.900	0.750
Hyperactivity-Impulsivity	0.880	0.715

The correlated two-factor model fit the adolescent data substantially better in descriptive terms than the one-factor model. Standardized two-factor loadings ranged from .631 to .945. The latent factor correlation was 0.825, showing that the two symptom domains were strongly related but not interchangeable. CR and AVE values supported convergent measurement evidence for both factors. Taken together, the results support use of the two-factor ASRS-CSV structure in this adolescent research sample.

Software and reproducibility

Data screening, descriptive statistics, and Cronbach's alpha were completed in IBM SPSS Statistics 26.0. Supplementary CFA, CR, and AVE analyses were conducted in R 4.5.0 with lavaan 0.6-20 using WLSMV estimation. No responses were missing for the analyzed items.
